# Magnetic Resonance Imaging–Guided Confirmatory Biopsy for Initiating Active Surveillance of Prostate Cancer

**DOI:** 10.1001/jamanetworkopen.2019.11019

**Published:** 2019-09-11

**Authors:** Rajiv Jayadevan, Ely R. Felker, Lorna Kwan, Danielle E. Barsa, Haoyue Zhang, Anthony E. Sisk, Merdie Delfin, Leonard S. Marks

**Affiliations:** 1 Department of Urology, David Geffen School of Medicine at University of California, Los Angeles; 2 Department of Radiology, David Geffen School of Medicine at University of California, Los Angeles; 3 Department of Pathology, David Geffen School of Medicine at University of California, Los Angeles

## Abstract

**Importance:**

Transrectal, ultrasonography-guided prostate biopsy often fails to disclose the severity of underlying pathologic findings for prostate cancer. Magnetic resonance imaging (MRI)–guided biopsy may improve the characterization of prostate pathologic results, but few studies have examined its use for the decision to enter active surveillance.

**Objective:**

To evaluate whether confirmatory biopsy findings by MRI guidance are associated with the risk of pathologic disease upgrading among patients with prostate cancer during active surveillance.

**Design, Settings, and Participants:**

This retrospective cohort study used prospectively obtained registry data from 332 men with prostate cancer of Gleason grade group (GG) 2 or lower who were referred for active surveillance at a large academic medical center from January 1, 2009, through December 31, 2017.

**Exposures:**

All confirmatory and follow-up biopsies were performed using MRI guidance with an MRI–ultrasonography fusion device. Patients underwent repeated MRI-guided biopsies every 12 to 24 months. At follow-up sessions, in addition to obtaining systematic samples, lesions seen on MRI were targeted and foci of low-grade prostate cancer were obtained again using tracking technology. Active surveillance was terminated with detection of at least GG3 disease or receipt of treatment.

**Main Outcomes and Measures:**

The primary outcome was upgrading to at least GG3 disease during active surveillance. Secondary outcomes were the associations of MRI lesion grade, prostate-specific antigen (PSA) level, PSA density, and biopsy method (targeted, systematic, or tracked) with the primary outcome.

**Results:**

Of 332 patients (mean [SD] age, 62.8 [7.6] years), 39 (11.7%) upgraded to at least GG3 disease during follow-up. The incidence of upgrading was 7.9% (9 of 114) when the confirmatory biopsy finding was normal, 11.4% (20 of 175) when the finding showed GG1 disease, and 23.3% (10 of 43) when the finding was GG2 disease (*P* = .03). Men with GG2 disease were almost 8 times more likely to upgrade during surveillance compared with those with normal findings but only among those with low PSA density (hazard ratio [HR], 7.82; 95% CI, 2.29-26.68). A PSA density of at least 0.15 ng/mL/mL was associated with increased risk of upgrading among patients with normal findings (HR, 7.21; 95% CI, 1.98-26.24) or GG1 disease (HR, 2.86; 95% CI, 1.16 to 7.03) on confirmatory biopsy. A total of 46% of pathologic disease upgrades would have been missed if only the targeted biopsy was performed and 65% of disease upgrades were detected only with tracked biopsy.

**Conclusions and Relevance:**

The findings suggest that confirmatory biopsy with MRI guidance is significantly associated with future disease upgrading of prostate cancer, especially when combined with PSA density, and should be considered as an appropriate entry point for active surveillance. Systematic and targeted biopsies were additive in detection of clinically significant cancers. Repeated biopsy at sites at which findings were previously abnormal (tracking biopsy) facilitated detection of cancers not suitable for continued active surveillance.

## Key Points

**Question:**

For men receiving active surveillance for low-risk prostate cancer, are confirmatory biopsy findings, when obtained with magnetic resonance imaging (MRI) guidance, associated with the subsequent risk of pathologic disease upgrading?

**Findings:**

In this cohort study of 322 patients, pathologic disease upgrading beyond low risk was significantly associated with findings from MRI-guided confirmatory biopsy for men undergoing active surveillance for prostate cancer. The 2 most important variables associated with subsequent pathologic disease upgrading were Gleason grade group and prostate-specific antigen density at the time of the confirmatory biopsy.

**Meaning:**

The findings suggest that an appropriate entry point for active surveillance for prostate cancer is confirmatory biopsy with MRI guidance and that normal confirmatory biopsy findings and a low prostate-specific antigen density may be associated with less risk of a pathologic disease upgrade compared with other results.

## Teaser

This cohort study examines the association of magnetic resonance imaging (MRI)−guided confirmatory prostate biopsy findings with the risk of pathologic disease upgrading during active surveillance for prostate cancer.

## Introduction

Active surveillance is currently the most rapidly growing management strategy for men with prostate cancer.^1,2,3,4^ The goal of active surveillance is to defer treatment for men with prostate tumors unlikely to cause mortality, thereby preventing the morbidity that active intervention often entails. Increasing adoption of active surveillance began in the 1990s, following the lead of programs such as those at Johns Hopkins University School of Medicine and the University of Toronto. Enrollment in the early active surveillance programs was primarily based on the biopsy findings of low-risk cancers, and discontinuing active surveillance was mainly based on a subsequent biopsy finding indicating increased risk.^5^ The strategy has been largely successful; few compliant individuals have experienced metastatic disease during active surveillance, and at present, most men who receive a diagnosis of with low-risk prostate cancer are treated expectantly.^4,6^

However, histologic upgrading of prostate cancer beyond the low-risk disease found initially has been reported in 30% of men during the first year of follow-up.^7^ Early disease upgrading likely indicates that the initial biopsy findings were inaccurate, which diminishes the efficiency of active surveillance and raises concern about the propriety of active surveillance. Therefore, accurate characterization of prostate pathologic findings from the start of active surveillance (and throughout follow-up) would be desirable.

Magnetic resonance imaging (MRI)–guided biopsy has been shown to help characterize pathologic findings more accurately than transrectal ultrasonography-guided (TRUS) biopsy, leading to improved detection of high-risk disease.^8,9,10,11,12,13^ However, use of this new biopsy method has not yet been fully evaluated among men undergoing active surveillance. We evaluated an active surveillance program started in January 1, 2009, approximately coincident with the advent of the MRI-guided biopsy technology and thus fundamentally different from older, larger programs. Most available data on active surveillance come from programs that began several decades ago, when prostate biopsy was performed systematically with ultrasonography guidance. In the present study, men diagnosed with low-risk cancers were examined with confirmatory biopsy and follow-up biopsy using MRI guidance exclusively.

## Methods

### Study Design

This cohort study used prospectively acquired data from a single-center registry for men with a new diagnosis of Gleason grade group (GG) 1 prostate cancer from January 1, 2009, through December 31, 2017. The GG system is a contemporary pathologic grading system of prostate cancer that incorporates total Gleason pattern scores: GG1 (Gleason score, ≤6), GG2 (Gleason score, 3 + 4 = 7), GG3 (Gleason score, 4 + 3 = 7), GG4 (Gleason score, 8); and GG5 (Gleason scores, 9 and 10). The University of California, Los Angeles Medical Institutional Review Board 2 approved this study. Written informed consent was obtained from all patients at enrollment. This study followed the Strengthening the Reporting of Observational Studies in Epidemiology (STROBE) reporting guideline.

In this study, the initial diagnostic biopsy was performed by various methods in community settings. Within 1 year of diagnosis, all men underwent confirmatory biopsy with multiparametric MRI guidance at the University of California, Los Angeles. Confirmatory biopsy and all follow-up biopsies were performed using an MRI-guided biopsy system (Artemis Biopsy System; Eigen Inc). The end point was a finding of at least GG3 disease during follow-up, which then excluded patients from active surveillance. All patients underwent software-templated 12-core systematic biopsy during each biopsy session; in addition, men with at least grade 3 lesions on multiparametric MRI underwent targeted biopsy, as previously described.^14,15,16^ After confirmatory biopsy, patients were monitored with a semiannual digital rectal examination and prostate-specific antigen (PSA) testing and underwent multiparametric MRI and follow-up biopsies every 12 to 24 months. Patients were excluded from the analytic cohort if they had not yet had at least 1 follow-up biopsy or if they received treatment, were followed up elsewhere, were lost to follow-up, withdrew consent, or died. We compared the patients who were excluded with the final analytic cohort.

### Imaging and Biopsy Methods

Multiparametric MRI of the prostate was performed with 1 of 4 Siemens 3-T magnets (Skyra, TrioTim, Magnetom VIDA, or Prisma) with pelvic phased-array coils. The MRI was repeated before each biopsy. All MRIs were interpreted by dedicated genitourinary radiologists (including E.R.F.). Before the Prostate Imaging Reporting and Data System (PI-RADS) was introduced, regions of interest (ROI) were graded using an in-house Likert scale that used measures similar to those used in PI-RADS, version 2 (v2). The University of California, Los Angeles scoring system is concordant with PI-RADSv2.^17^ Beginning in late 2014, ROIs were graded using the PI-RADSv2 scoring system. The ROIs were contoured by radiologists using the ProFuse software (Eigen Inc) and were then transferred to the fusion device.

**Figure 1.**
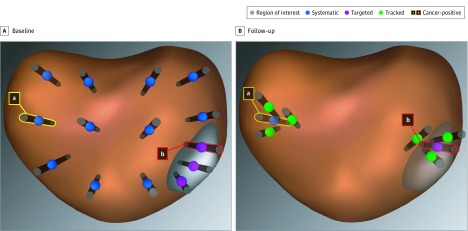
Systematic, Targeted, and Tracked Prostate Biopsy Schematic Biopsy approaches shown on reconstructed images from magnetic resonance imaging–ultrasonography fusion device. A, Region of interest with 3 targeted biopsy cores, 1 containing cancer tissue (b). Systematic biopsy with 1 biopsy core containing cancer tissue (a). B, Follow-up with tracked biopsy cores from the cancerous site within (b) and distant from (a) the region of interest. Tracked biopsies were placed to circumscribe a previously abnormal site.

Samples were obtained by targeted biopsy from each ROI, with at least 1 core obtained within every 3 mm of the longest axis of the ROI, as described elsewhere.^18^ All biopsy coordinates were registered and stored within the fusion system. Samples from the core coordinates that were found to have cancer cells were obtained again with tracked biopsy during follow-up to monitor for disease progression (Figure 1).^19^ All biopsies were performed by a coauthor (L.S.M.).

### Outcomes

The main outcome of interest was pathologic upgrading of prostate cancer to at least GG3 disease.^20,21,22^ Upgrade-free survival was defined as the time from the confirmatory biopsy to the last eligible biopsy. For patients found to have pathologic disease upgrading, upgrade-free survival was the date of upgrade identification. For patients without pathologic disease upgrading, this was the date of the last biopsy during active surveillance in the study period. For patients who elected to undergo treatment without having at least GG3 disease identified, end date was the last biopsy treatment. Pathologic upgrading by biopsy method (ie, targeted biopsy vs systematic biopsy or tracked vs nontracked biopsy) was also determined.

### Statistical Analyses

The association between pathologic findings and clinical characteristics was evaluated with χ^2^ tests (or Fisher exact tests when necessary) and *t* tests (or Wilcoxon rank sum tests when necessary). For the multivariable analysis, an upgrade-free survival analysis was conducted to calculate adjusted hazard ratios (HRs) for confirmatory biopsy pathologic findings. Patients were censored if they did not experience upgrading during the study period, were followed up elsewhere, died during the study period, or were lost to follow-up. Some patients received treatment without identification of at least GG3 disease upgrading, which precluded the identification of at least GG3 disease while undergoing active surveillance. Therefore, a multivariable competing risk analysis was conducted with receipt of treatment designated as the competing risk. Several confirmatory biopsy pathologic results and patient characteristics were chosen a priori for model inclusion: patient age, PI-RADSv2 score, PSA level, prostate volume, PSA density (<0.15 vs ≥0.15 ng/mL/mL), maximum cancer core length, and percent tumor involvement. We conducted separate competing risk models for each variable as well as a full model with all the variables included. Finally, we explored potential factors associated with confirmatory GG scores. For all models, GG scores on confirmatory biopsy were included, and we tested for the proportionality assumption. All tests were 2-sided with an α of .05, and all statistical analyses were performed by 2 of us (L.K. and H.Z.) using SAS, version 9.4 software (SAS Institute).

## Results

**Figure 2.**
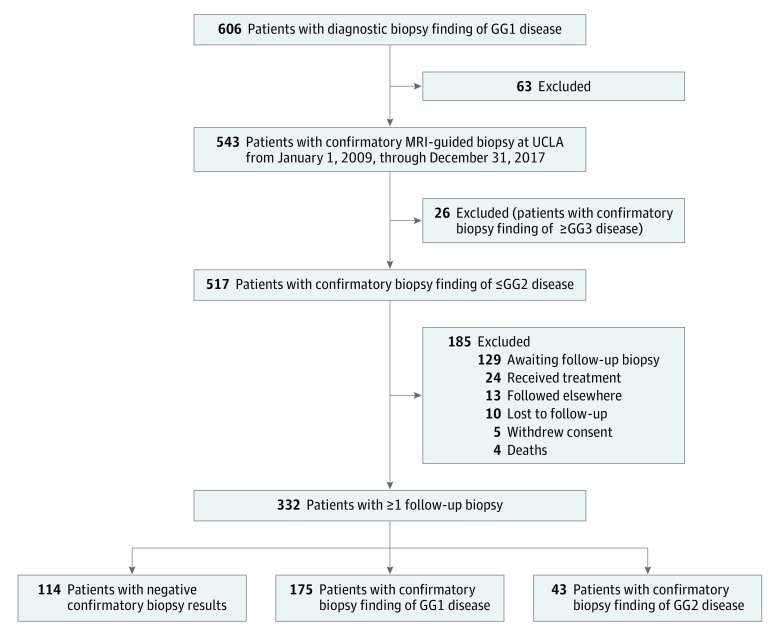
Flow Diagram of Participants GG indicates Gleason grade group; GG1, Gleason score of 6 or lower; GG2, Gleason score of 3 + 4 = 7; GG3, Gleason score of 4 + 3 = 7; MRI, magnetic resonance imaging; and UCLA, University of California, Los Angeles.

Of the 606 patients with GG1 disease who were enrolled into the acute surveillance registry at the University of California, Los Angeles, 517 had GG2 or lower on confirmatory biopsy. Of 332 patients (mean [SD] age, 62.8 [7.6] years) in the total cohort after exclusions, 114 had normal findings on confirmatory biopsy, 175 had GG1 disease, and 43 had GG2 disease. All 332 patients had at least 1 follow-up biopsy and comprise the analytic cohort (Figure 2), and 185 patients who had GG2 disease or lower on confirmatory biopsy were excluded, including 129 patients who were still awaiting a follow-up biopsy. This group of excluded patients was compared with the analytic cohort and was not found to differ in GG grade on confirmatory biopsy, age, PSA level, family history of prostate cancer, educational level, or income.

Patient characteristics at time of confirmatory biopsy are presented in Table 1. The median follow-up time for all patients was 3.9 years (range, 0.3-8.5 years). The median time between follow-up biopsies was 1.2 years (range, 1.0-2.0 years), and the median number of follow-up biopsies was 2 (range, 1-6 biopsies). Of the 332 patients, 229 (69.0%) had at least 1 ROI detected on multiparametric MRI before the confirmatory biopsy was performed; 90 of 229 (39.3%) of those with an ROI had at least 1 grade 4 or 5 lesion, and 139 of 229 (60.7%) had grade 3 ROIs. A median of 4 cores (range, 3-11 cores) was obtained from each ROI during each biopsy session.

**Table 1.** Patient Characteristics by Confirmatory Biopsy Pathologic Findings^a^

Variable	Total (N = 332)	Negative (n = 114)	GG1 (n = 175)	GG2 (n = 43)	*P* Value
Age, mean (SD), y	62.8 (7.6)	61.9 (7.5)	63.3 (7.7)	63.1 (7.1)	.32
Race/ethnicity^b^					
Non-Hispanic white	257 (77.4)	91 (79.8)	137 (78.3)	29 (67.4)	.23
Nonwhite	75 (22.6)	23 (20.2)	38 (21.7)	14 (32.6)	
Family history of prostate cancer					
Yes	80 (24.1)	28 (24.6)	44 (25.1)	8 (18.6)	.66
No	252 (75.9)	86 (75.4)	131 (74.9)	35 (81.4)	
Palpable abnormality					
Yes	12 (3.6)	3 (2.6)	7 (4.0)	2 (4.7)	.99^c^
No	117 (35.2)	27 (23.7)	69 (39.4)	21 (48.8)	
Reasons for end of follow-up					
Received treatment^d^	72/88 (21.7)	11/17 (9.6)	43/50 (24.6)	18/21 (41.9)	NA
Followed up elsewhere	11/88 (3.3)	4/17 (3.5)	5/50 (2.9)	2/21 (4.7)	
Nonprostate cancer death	3/88 (0.9)	1/17 (0.9)	1/50 (0.6)	1/21 (2.3)	
Lost to follow-up	2/88 (0.6)	1/17 (0.9)	1/50 (0.6)	0/21	
PI-RADSv2 score^e^					
No target	103 (31.0)	32 (28.1)	54 (30.9)	17 (39.5)	.44
3	139 (41.9)	52 (45.6)	71 (40.6)	16 (37.2)	
4	73 (22.0)	28 (24.6)	39 (22.3)	7 (16.3)	
5	16 (4.8)	2 (1.8)	11 (6.3)	3 (7.0)	
Prostate volume, median (IQR), mL	48.0 (34.3-70.5)	54.1 (37.0-76.2)	45.8 (33.9-67.7)	46.0 (34.0-63.9)	.29^f^
PSA level, median (IQR), ng/mL	4.7 (2.5-7.0)	4.6 (2.3-7.4)	4.8 (2.8-6.8)	5.4 (2.3-7.3)	.90^f^
PSA density, median (IQR), ng/mL/mL	0.08 (0.05-0.14)	0.08 (0.05-0.12)	0.08 (0.06-0.14)	0.09 (0.06-0.14)	.33^f^
Duration of active surveillance, median (IQR) [range], y	3.9 (2.6-5.9) [0.2-8.5]	5.4 (3.7-6.5) [1.1-8.4]	3.7 (2.3-5.4) [0.3-8.5]	2.3 (1.0-3.6) [0.2-8.4]	<.001^f^
Maximum cancer core length, median (IQR), mm	2.0 (1.0-4.0)	NA	2.0 (1.0-4.0)	4.0 (2.0-6.0)	.001^f^
Tumor involvement, median (IQR), %	20.0 (5.0-30.0)	NA	15.0 (5.0-30.0)	25.0 (15.0-40.0)	.001^f^
Free PSA, median (IQR), %	18.0 (14.0-24.0)	20.0 (16.0-25.2)	18.0 (13.0-24.2)	15.0 (11.0-18.0)	.001^f^

Abbreviations: GG1, Gleason score of 6 or lower; GG2, Gleason score of 3 + 4 = 7; IQR, interquartile range; NA, not applicable; PI-RADSv2, Prostate Imaging and Reporting Data System, version 2; PSA, prostate-specific antigen level.

^a^ Data are presented as number or number/total number (percentage) of patients unless otherwise indicated.

^b^ Race/ethnicity was self-reported. Nonwhite race includes African American, Hispanic, Asian, and other.

^c^ Fisher exact test.

^d^ Radical prostatectomy (n = 35), radiation therapy (n = 18), cryoablation (n = 8), high-intensity focused ultrasound (n = 4), laser interstitial thermal therapy (n = 2), and other (n = 4).

^e^ If PI-RADSv2 score was unavailable, University of California, Los Angeles prostate lesion score was used.

^f^ Wilcoxon rank sum test.

### Overall Upgrade Incidence (All Biopsy Methods Combined)

There were 39 patients (11.7%) with disease upgraded to at least GG3 during the study period (eTable 1 in the Supplement). The incidence of pathologic disease upgrading varied significantly by pathologic findings at confirmatory biopsy: 9 of 114 (7.9%) with normal findings had a disease upgrade, 20 of 175 (11.4%) with GG1 disease had an upgrade, and 10 of 43 (23.3%) with GG2 disease had an upgrade (*P* = .03).

The annual reclassification rate to at least GG3 disease was 1.5% among patients with normal confirmatory biopsy findings, 3% among those with GG1 disease on confirmatory biopsy, and 9.9% among those with GG2 disease on confirmatory biopsy. The median upgrade-free survival after confirmatory biopsy was longer for men with normal findings (2.8 years; interquartile range, 2.0-3.3 years) compared with those who had GG1 disease (1.9 years; interquartile range, 1.0-3.4 years) and those who had GG2 disease (1.4 years; interquartile range, 1.0-2.2 years) (*P* = .05). The median time that patients underwent active surveillance after the confirmatory biopsy was longer for those with normal findings (5.4 years; interquartile range, 3.6-6.5 years) compared with those with GG1 disease (3.7 years; interquartile range, 2.4-5.3 years) and those with GG2 disease (2.2 years; interquartile range, 1.0-3.5 years) (*P* < .001).

The following variables were significantly associated with pathologic disease upgrading to at least GG3 disease on univariate analysis: confirmatory biopsy pathologic findings, PSA level more than 10 ng/mL, percent free PSA level less than 10%, PSA density of at least 0.15 ng/mL/mL, and a percentage of tumor involvement at least 50% of biopsy core (eTable 2 in the Supplement). All these variables, along with maximum cancer core length, were included in the multivariable analysis. However, after entering all these variables, the model was unstable and produced large, unreliable 95% CIs (eg, upper limit of 127.5). We therefore limited variable inclusion to the variables in the a priori list that also had *P* < .10 in the univariate analysis.

**Figure 3.**
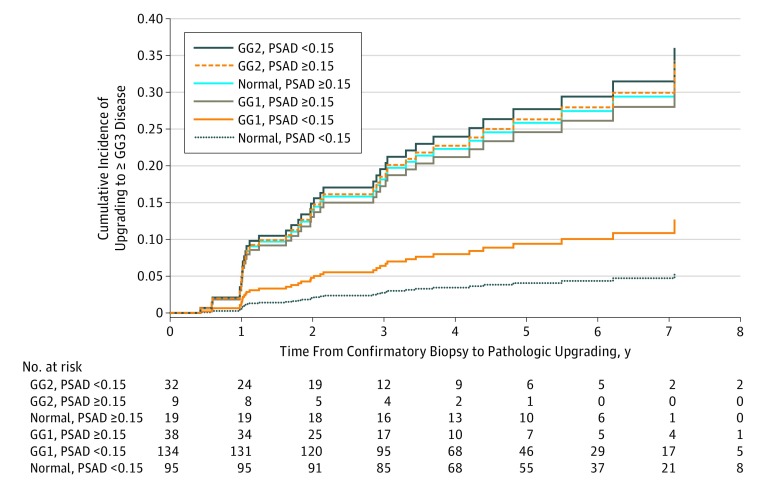
Competing Risk Analysis for Pathologic Disease Upgrading by Confirmatory Biopsy and Prostate-Specific Antigen Density (PSAD) Receipt of treatment before indication of upgrading was designated as the competing risk. GG indicates Gleason grade group; GG1, Gleason score of 6 or lower; GG2, Gleason score of 3 + 4 = 7; and GG3, Gleason score of 4 + 3 = 7.

Forty-five patients elected treatment without having at least GG3 disease identified, precluding the identification of GG3 disease or higher for these men while they underwent active surveillance. The resulting competing risk analysis, with treatment designated as the competing risk, revealed an association of confirmatory biopsy pathologic findings and PSA density with upgrading to GG3 disease or higher (Table 2). The association of GG grade with disease upgrading, however, was modified by PSA density as shown by significance at varying levels of the interaction term of GG grade on confirmatory biopsy and PSA density. Patients with GG2 disease on confirmatory biopsy had an almost 8-fold greater rate of upgrading compared with those with normal confirmatory biopsy findings (HR, 7.82; 95% CI, 1.29-26.68) and a more than 3-fold greater rate compared with those with GG1 disease on confirmatory biopsy (HR, 3.3; 95% CI, 1.3-8.4) but only among patients with low PSA density. There was no significant difference between GG1 disease and normal findings on confirmatory biopsy among patients with low PSA density or between any of the GG groups among patients with high PSA density. Patients with a high PSA density had greater HRs than those with low PSA density among patients with normal findings (HR 7.21; 95% CI, 1.98-26.24) or GG1 disease (HR 2.86; 95% CI, 1.16-7.03) on confirmatory biopsy. Figure 3 shows the cumulative incidence function from the competing risk analysis for pathologic upgrading to at least GG3 disease stratified by confirmatory biopsy pathologic findings and PSA density.

**Table 2.** Competing Risk Analysis of Variables Associated With Upgrading^a^

Variable	Univariate Analysis		Multivariate Analysis	
	Hazard Ratio (95% CI)	*P* Value	Hazard Ratio (95% CI)	*P* Value
Age at diagnosis, y	1.02 (0.97-1.08)	.35	NA	NA
PI-RADSv2 score				
Normal	1 [Reference]	.11		NA
3	0.51 (0.22-1.22)		NA	
4	1.07 (0.48-2.43)		NA	
5	0.10 (0.01-1.13)		NA	
PSA level, ng/mL	1.05 (1.00-1.11)	.06	NA	NA
Prostate volume on MRI, mL	1.00 (0.99-1.01)	.90	NA	NA
GG^b^				
Normal	1 [Reference]	<.001	NA	NA
GG1	14.73 (4.77-45.46)		NA	
GG2	14.44 (4.20-49.61)		NA	
PSA density level, ≥0.15 vs <0.15 ng/mL/mL	1.74 (0.82-3.69)	.15	NA	.003
MCCL, ≥6 vs <6 mm	0.80 (0.25-2.53)	.71	NA	NA
Percent cancer, ≥25% vs <25%	1.02 (0.40-2.62)	.97	NA	NA
GG and PSA density interaction^c^				
GG1 vs normal		NA		.16
<0.15 ng/mL/mL	NA		2.38 (0.77-7.34)	
≥0.15 ng/mL/mL	NA		0.94 (0.31-2.84)	
GG2 vs normal				
<0.15 ng/mL/mL^c^	NA		7.82 (2.29-26.68)	
≥0.15 ng/mL/mL	NA		1.02 (0.19-5.57)	
GG2 vs GG1				
<0.15 ng/mL/mL^c^	NA		3.29 (1.29-8.36)	
≥0.15 ng/mL/mL	NA		1.08 (0.21-5.63)	
PSA density ≥0.15 vs <0.15 ng/mL/mL				
Normal^d^	NA		7.21 (1.98-26.24)	
GG1^d^	NA		2.86 (1.16-7.03)	
GG2	NA		0.94 (0.18-4.99)	

Abbreviations: GG, Gleason grade group; GG1, Gleason score of 6 or lower; GG2, Gleason score of 3 + 4 = 7; MCCL, maximum cancer core length; MRI, magnetic resonance imaging; NA, not applicable; PI-RADSv2, Prostate Imaging and Reporting Data System, version 2; PSA, prostate-specific antigen.

^a^ Receipt of treatment before detection of pathologic disease upgrading was designated as the competing risk.

^b^ GG2 vs GG1: univariate model hazard ratio, 0.98; 95% CI, 0.23-4.13.

^c^ Hazard ratios for each level of GG and PSA density in the multivariate model.

^d^ Significant hazard ratio.

### Targeted vs Systematic Biopsy

Forty-one percent of grade GG3 of higher lesions were detected by targeted biopsy only, 45% by systematic biopsy only, and 14% by both methods, indicating a high-level of disagreement of tumor detection by biopsy method (κ, –0.77; 95% CI, –0.94 to –0.60). Among 28 men with both targeted biopsy and systematic biopsy findings at the time of disease upgrade, the rate of disease upgrade detection was similar: 43% of disease upgrades were detected only by targeted biopsy, and 46% were detected only by systematic biopsy. Thus, if only 1 biopsy method was implemented, at least 43% of disease upgrades would have been missed.

### Tracked Biopsy

Thirty-four of the 39 patients (87%) who had disease upgrade to at least GG3 disease had a tracked biopsy at the time of upgrade detection; 23 of these 34 upgrades (68%) to grade GG3 of higher disease were found on tracked biopsy, 22 upgrades (65%) were found on only tracked biopsy, and 1 (3%) was detected by both tracked and nontracked biopsy. Of the tracked biopsies, 12 (35%) were within an ROI (targeted biopsy), 14 (41%) were outside an ROI (systematic biopsy), and 8 (24%) had at least 1 tracked core in both targeted and systematic biopsies. Thus, tracked biopsies outside ROIs revealed just as many upgrades as tracked biopsies within ROIs.

### Active Treatment

During this study, 29 of 39 patients who had disease upgrades to grade GG3 or higher (74%) and 45 of 293 patients without disease upgrade (15%) received treatment. Median time from the confirmatory biopsy to treatment was 2.4 years (interquartile range, 1.3-3.7 years). Thirty-five patients underwent radical prostatectomy, and 18 were treated with radiotherapy; others were treated with focal therapies (Table 1).

## Discussion

### Upgrading During Active Surveillance

We aimed to define the value of the MRI-guided confirmatory biopsy for evaluating the risk of pathologic disease upgrading to grade GG3 or higher prostate cancer for men undergoing active surveillance. The GG3 disease was chosen as a discrete end point because of widespread agreement that patterns exceeding GG2 disease are beyond suitability for active surveillance.^20,22^ Although GG3 disease does not necessarily equate with increased mortality, the increased risk of metastatic disease associated with that pathologic grade generally precludes active surveillance.^23^ The patients comprised a large active surveillance cohort in which all confirmatory and follow-up biopsies were performed exclusively using MRI guidance technology. The data suggest that normal confirmatory biopsy findings, when performed using MRI-guided biopsy, are associated with long-term protection against pathologic disease upgrading to grade GG3 and that confirmatory biopsy findings of GG2 disease are associated with a 3.5-fold greater rate of upgrading to GG3 disease compared with normal confirmatory biopsy findings. We also found that the combination of targeted and systematic biopsies was additive for the detection of pathologic disease upgrading and that many upgrades were detected only by repeated testing of previously positive sites (ie, tracked biopsy).

### Confirmatory MRI-Guided Biopsy

A normal confirmatory biopsy finding, when obtained using conventional TRUS methods, is reportedly associated with a 50% to 70% risk reduction for pathologic disease upgrading.^24,25^ However, the prognostic value of confirmatory biopsy performed with MRI guidance, which is associated with improved detection of clinically significant disease, has been the subject of few studies.^26^ Our data revealed that after MRI-guided confirmatory biopsy qualified a patient for active surveillance, the overall risk of developing serious disease (GG3 disease) was approximately 12%. In this cohort, the risk was less than 8% for a patient with normal confirmatory biopsy findings, 11% for confirmatory biopsy findings of GG1 disease, and 23% for confirmatory biopsy findings of GG2 disease. These data suggest that confirmatory biopsy with MRI guidance provides a more accurate risk assessment than when the confirmatory biopsy is performed by the conventional TRUS biopsy method. Furthermore, when future risk is low (ie, normal MRI findings or low PSA density), the frequency of routine follow-up biopsy might be diminished.

At present, enrollment in active surveillance is often recommended at the time of an initial diagnosis, and confirmatory biopsy and follow-up biopsy are performed at highly variable intervals.^27,28^ The present data appear to be in favor of an MRI-guided confirmatory biopsy within 1 year of diagnosis to verify a patient’s eligibility for active surveillance.^29^ If confirmed, these data would suggest that active surveillance should formally begin after an MRI-guided confirmatory biopsy has been performed, providing information to help make a rational decision about risks of eventually requiring active intervention. For example, a 55-year-old man with long life expectancy who is found to have GG2 disease on MRI-guided confirmatory biopsy may elect active treatment at that time, considering his substantial risk of upgrading to GG3 disease. A 75-year-old man might decide otherwise. Subclassification of patients by the volume of Gleason pattern 4 (GG4) or genomic analyses may stratify risk even further.^30,31^ Still lacking, however, are data to show when, if ever, the risk of disease upgrading ends and follow-up can be discontinued altogether.

In an earlier study of 182 men, Bloom et al^26^ reported on the prognostic value of normal confirmatory biopsy findings when biopsy is performed with MRI guidance. Our results among 332 men corroborate their central finding that confirmatory biopsy pathologic findings and PSA density are associated with disease upgrading. Moreover, the present study includes a definition of pathologic disease upgrading (GG3), a structured protocol for follow-up biopsy regardless of clinical measures, and an expanded sample size.

### Combination Biopsy

If performed exclusively, targeted and systematic biopsy would have each failed to detect a large portion of clinically significant tumors among men with ROIs during active surveillance: 41% of GG3 or higher disease upgrades would have been missed if targeted biopsy was not performed and 45% if systematic biopsy was not performed. Similar findings were seen in the recently published Improvement in the Detection of Aggressive Prostate Cancer by Targeted Biopsies Using Multiparametric MRI Findings (MRI-FIRST) and the Prospective Assessment of Image Registration in the Diagnosis of Prostate Cancer (PAIREDCAP) trials.^18,32^ As in our study, patients with ROIs in the MRI-FIRST trial underwent both systematic and targeted biopsy, which allowed for investigation of the added value of systematic and targeted biopsy findings within the same patient. In the MRI-FIRST trial, the detection of GG2 or higher tumors was improved when both biopsy techniques were combined. In the MRI-Targeted or Standard Biopsy for Prostate-Cancer Diagnosis (PRECISION) trial, men with a visible lesion by MRI underwent targeted biopsy only, and thus the value of combining targeted and systematic biopsy findings could not be determined.^12^

An analysis by Frye et al^33^ of patients undergoing active surveillance at the National Cancer Institute also found that the combination of systematic and targeted biopsy should be used during follow-up, given that 30% of pathologic disease upgrades were identified by systematic biopsy alone. The combination of both biopsy techniques among patients with ROIs has also been recently advocated by others, particularly for high-risk patients.^16,34,35^ Together, these findings suggest that although MRI-targeted biopsy may be associated with improved detection of clinically significant disease, it does not yet obviate the need for systematic biopsy.

### Biopsy Site Tracking

In this study, tracked biopsy helped detect upgrades to GG3 or higher disease. Sixty-eight percent of patients who had upgrades to GG3 or higher disease had an upgrade detected tracked biopsy. The tracking utility was helpful both within MRI-visible ROIs and at abnormal sites on systematic biopsy outside ROIs. Tracking biopsy detected substantially more disease upgrades than did nontracked biopsy. Among the 34 men who had both tracked and nontracked cores obtained during biopsy in which disease was upgraded, upgrading was detected only by tracked biopsy in 22 men (65%).

Other methods of lesion targeting are available (eg, direct in-bore targeting or ultrasonography-guided cognitive targeting), but with an image-fusion device software is provided for storage of lesion location for future recall. Studies previously have shown that tracked biopsies are able to return to a prior biopsy site within a few millimeters and are useful in the detection of upgrading.^16,19,36,37^ Thus, the value of tracked biopsy, which has been studied little compared with targeting biopsy, deserves further evaluation in prospective trials.

In this study, 14 men (36%) who had disease upgrade to at least GG3 disease had normal MRI findings before the confirmatory biopsy. Pathologic disease upgrades were detected only by systematic biopsy for these patients. The negative predictive value for multiparametric MRI detecting at least GG3 disease upgrades was 86%. These negative predictive values were within the range of those reported in other large cohort studies and reveal that a significant percentage of men with normal MRI findings were found to have clinically significant disease.^38^ Our findings do not support the use of normal MRI findings to obviate follow-up biopsies in all men undergoing active surveillance but suggest that systematic biopsy should still be considered in the absence of a grade 3 or higher lesion, especially if the PSA density is elevated. The reason that MRI sometimes fails to detect clinically significant tumors has not been fully elucidated. Recent reports show that certain morphologies of prostate cancer, such as mucinous adenocarcinoma and cribriform variants, are often not apparent on diffusion-weighted imaging.^39,40^ Another possible explanation is that small-volume tumors that are not readily visible on MRI may still have large surface areas that are more likely to be detected by systematic biopsy. Regardless of the reason, the negative predictive value suggests that follow-up active surveillance biopsy should continue in men with normal MRI findings.

### Limitations

This study has several limitations. All systematic biopsies were performed using a template proposed by the fusion device, which further differentiates systematic results of conventional TRUS biopsy from present systematic results. Unlike conventional TRUS biopsy, which requires the clinician to cognitively resolve prostate anatomy, this software template is designed to improve systematic sampling. Thus, the sensitivity of systematic biopsy in this cohort may have exceeded that of conventional systematic biopsy performed without software guidance.^41^ Furthermore, results reported here are from an expert center, where thousands of MRI-guided biopsies have been performed by a cohesive team, including clinicians trained in urology, radiology, pathology, and biomedical engineering, during the past decade. However, this high-volume experience with MRI guidance may also prevent these results from being generalizable to less-experienced clinicians. Another limitation to this study is the small cohort size and short follow-up duration. However, the cohort size and length of follow-up compare favorably with other investigations of MRI-guided biopsy. Moreover, all patients had at least 2 consecutive biopsies during a follow-up of nearly 4 years.

## Conclusions

The findings suggest that men with apparent low-risk prostate cancer who undergo confirmatory biopsy with MRI or ultrasonography guidance have improved risk assessment compared with conventional TRUS biopsy. Combination biopsy (targeted and systematic) was a more sensitive method of detecting tumors not suitable for continued surveillance than either method alone. Repeated biopsy of previous positive coordinates (tracking) may be an important means of detecting disease upgrades and deserves further study. Taken together, the results suggest that confirmatory biopsy by MRI guidance is associated with improved individual risk assessment and may serve as an appropriate entry point for active surveillance.

## Notes

Supplement. **eTable 1.** Upgrading Summary by Confirmatory Biopsy Pathology **eTable 2.** Patient Characteristics at Confirmatory Biopsy by Upgrading Status (N = 332)
