# Supplementary material for: Magnetic Resonance Imaging–Guided Confirmatory Biopsy for Initiating Active Surveillance of Prostate Cancer
Source: JAMA Netw Open. 2019 Sep 11;2(9):e1911019. doi: 10.1001/jamanetworkopen.2019.11019 (PMC6739900; doi:10.1001/jamanetworkopen.2019.11019)
Supplement: Supplement. — eTable 1. Upgrading Summary by Confirmatory Biopsy Pathology eTable 2. Patient Characteristics at Confirmatory Biopsy by Upgrading Status (N = 332) [file jamanetwopen-2-e1911019-s001.pdf]

Supplementary Online Content

Jayadevan R, Felker ER, Kwan L, et al. Magnetic resonance imaging–guided confirmatory biopsy for initiating active surveillance of prostate cancer. *JAMA Netw Open*. 2019;2(9):e1911019. doi:10.1001/jamanetworkopen.2019.11019

eTable 1. Upgrading Summary by Confirmatory Biopsy Pathology

eTable 2. Patient Characteristics at Confirmatory Biopsy by Upgrading Status (N = 332)

This supplementary material has been provided by the authors to give readers additional information about their work.

© 2019 Jayadevan R et al. *JAMA Network Open.*

eTable 1. Upgrading Summary by Confirmatory Biopsy Pathology

	**Total**<br>N=332	**Negative**<br>n=114	**GG1**<br>n=175	**GG2**<br>n=43
≥GG3 Upgrading, No. (%)	39/332 (11.7)	9/114 (7.9)	20/175 (11.4)	10/43 (23.3)
1st follow-up biopsy	21/146	3/53	11/70	7/23
2nd follow-up biopsy	10/110	5/40	3/59	2/11
3rd follow-up biopsy	2/51	0/15	2/29	0/7
4th follow-up biopsy	4/14	1/5	2/8	1/1
5th follow-up biopsy	1/8	0/1	1/6	0/1
6th follow-up biopsy	1/3	0/0	1/3	0/0
Years from confirmatory biopsy to ≥GG3 upgrading, median (IQR)[a]	2.0 (1.0, 3.3)	2.8 (2.0, 3.3)	1.9 (1.0, 3.4)	1.4 (1.0, 2.2)

a. Median test p=0.11
Abbreviations: GG, Gleason Grade Group

© 2019 Jayadevan R et al. *JAMA Network Open.*

eTable 2. Patient Characteristics at Confirmatory Biopsy by Upgrading Status (N=332)

	No upgrading	Upgrading to ≥GG3	
	n=293	n=39	
Age, mean (SD), y	62.6 (7.7)	64.2 (7.0)	0.22
Race, No. (%)[a]			
Non-Hispanic white	66 (23)	9 (23)	0.94
Non-white	227 (77)	30 (77)	
Family History of Prostate Cancer, No. (%)			
No	225 (77)	27 (69)	0.30
Yes	68 (23)	12 (31)	
Palpable Abnormality, No. (%)			
No	105 (36)	12 (31)	0.62[a]
Yes	10 (3)	2 (5)	
Confirmatory Pathology, No. (%)			
Negative	105 (36)	9 (23)	0.03
GG1	155 (53)	20 (51)	
GG2	33 (11)	10 (26)	
Reasons for End of Follow-up, No. (%)	n=58	n=35	
Non-prostate cancer death	2 (1)	1 (3)	0.40[b]
Followed elsewhere	9 (3)	2 (5)	
Lost to Follow-up	2 (1)	3 (8)	
Received treatment	45 (15)	29 (74)	
MRI Grade[c], No. (%)			
No Target	89 (31)	14 (36)	0.28
3	127 (43)	12 (31)	
4	62 (21)	12 (31)	
5	15 (5)	1 (3)	
Prostate volume, median (IQR), mL	50.0 (34.0, 71.0)	42.0 (36.0, 52.8)	0.36[d]
PSA, median (IQR), ng/mL	4.6 (2.4, 7.0)	5.5 (4.0, 8.2)	0.03[d]
PSA density, median (IQR), ng/mL/mL	0.08 (0.05, 0.12)	0.12 (0.07, 0.19)	0.002[d]
Years in active surveillance	4.1 (2.9, 6.1)	2.0 (1.0, 3.2)	<0.001[d]
Maximum cancer core length, median (IQR), mm	2.0 (1.0, 4.0)	3.1 (1.5, 5.0)	0.12[d]
% Tumor involvement, median (IQR)	15.0 (5.0, 30.0)	27.5 (15.0, 40.0)	0.04[d]
% Free PSA, median (IQR)	19.0 (14.0, 25.0)	14.0 (10.5, 17.0)	<0.001[d]

a. Non-white race includes African Americans, Hispanics, Asians, and “other”
b. Fisher’s exact test
c. If PI-RADS v2 score unavailable, UCLA prostate lesion score was used
d. Median test
Abbreviations: GG, Gleason Grade Group; PSA, prostate specific antigen

© 2019 Jayadevan R et al. *JAMA Network Open.*
